# The Comparison Between the Mutated HuIFN-β 27-101 and the Wild Type Interferon β: the Comprehensive In Silico Study to Evaluate the Effect of Mutations on IFN-β

**DOI:** 10.15171/apb.2019.074

**Published:** 2019-10-24

**Authors:** Sayed Sharif Balkhi, Zohreh Hojati

**Affiliations:** Division of Genetics, Department of Biology, Faculty of Sciences, University of Isfahan, Postal Code: 81746-73441 Isfahan, Iran.

**Keywords:** Interferon Beta, Molecular Docking, Insilico, IFNAR, mHuIFN-β-27-101

## Abstract

***Purpose:*** Interferon beta (IFN-β) is used to combat multiple sclerosis (MS) disease. Creating R27T and V101F mutations (mHuIFN-β-27 and mHuIFN-β-101) is one of the tasks performed to improve human interferon beta (HuIFN-β) half-life, function and expression. In this work, the impact of R27T and V101F mutations in recombinant IFN-β on its binding to interferon receptors were studied by molecular docking.

***Methods:*** This work was performed through in silico study. The simulation of mutation was performed using the online Rosetta Backrub software and checked using server verify3D. Comparison of access to the solvent of the amino acids in the structures created was performed using the asaview online server. Also, the effect of mutations on the fold of the protein was reviewed by the online HOPE server. The molecular docking was performed between HuIFN-β and the external region of IFNAR receptor using the online ClusPro2 protein-protein docking server.

***Results:*** The comparison of the values of the negative binding energy (ΔGbind) obtained from protein-protein molecular docking between IFNAR receptor and HuIFN-β, mHuIFN-β-27, mHuIFN-β-101 and mHuIFN-β-27-101 ligands did not show a significant difference, and these differences do not see any meaningful relationship between them (P > 0.9999).

***Conclusion:*** Regarding these results, it can be concluded that these mutations do not have a negative effect on the composition of the complex rHuIFN-β/IFNAR. So, they do not interfere with the binding of the IFN-β to the receptor. It is concluded that the quality of the rHuIFN-β is improved by introducing these two mutations.

## Introduction

Multiple sclerosis (MS) is a chronic disorder of the central nervous system, which, as a result of autoimmune attacks on the neural tissue, classically damages the macular degeneration of the axon and leads to the formation of polygonal lesions in the white matter.^1-3^ MS is phenotypically divided into several groups, the most common of which is the relapsing-remitting multiple sclerosis (RRMS).^3^ Despite the recent advances in the treatment of MS, interferon beta (IFN-β) still has the highest levels of prescription drug among immunosuppressive drugs for RRMS patients.^1^

**Figure 1:**
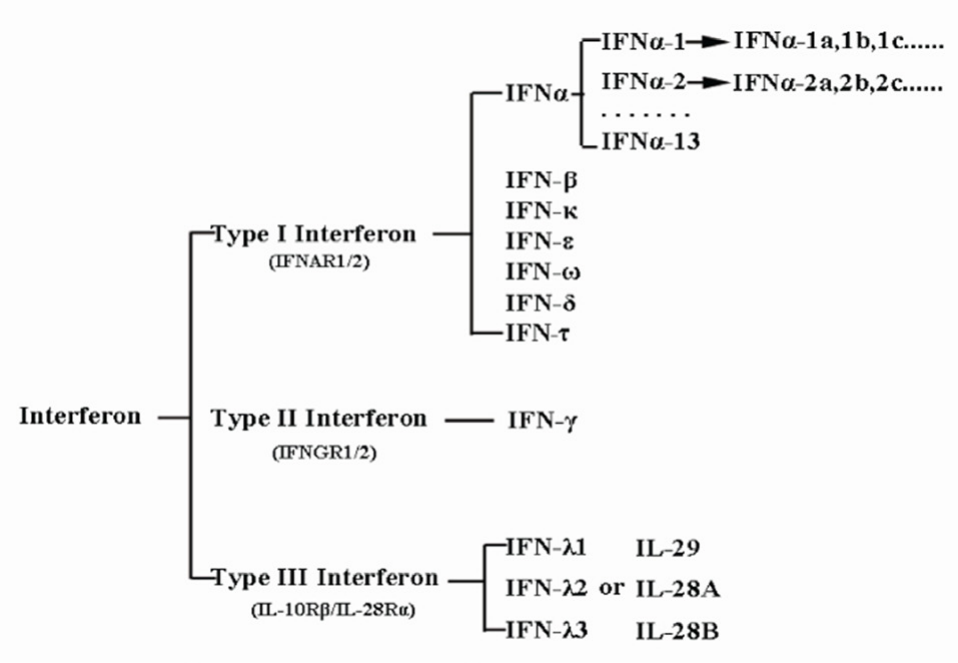
Categorization of different types of interferons. Based on the receptor type on the cell surface that transmits the signal inside the cell the interferons are divided into three major classes and each them includes subtypes with the exception of type II interferon.

**Figure 2:**
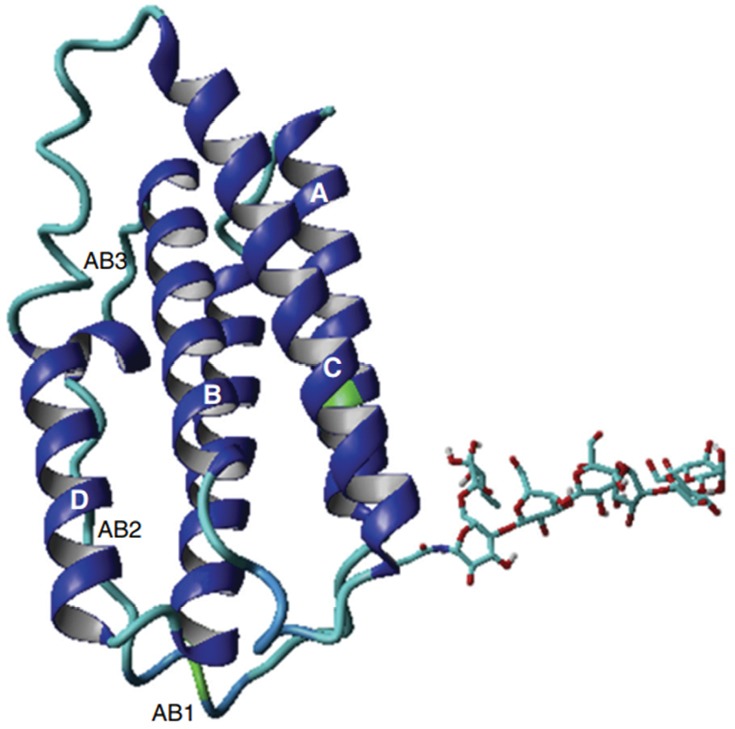
The ribbon structure of human interferon beta includes five alpha-helices A (A1, A2), B, C (C1, C2), D, and E also three loops, AB (AB1-Ab3), BC, CD (CD1, CD2). Loops AB1, AB2, and AB3 are labeled. The carbohydrate chains are represented beside the molecule.

Interferons were discovered for the first time due to their antiviral properties in 1957 by Isaacs and Lindenmann.^4^ Today, a wide range of functions is evident in diverse biological fields such as defense against microbial infections, antiviral, anticancer, autoimmune, inflammatory, immunization, proliferation, differentiation and survival of the cell.^4-6^ These small messenger molecules (kDa 30>) are secreted by various cells of the vertebrates in response to various stimuli that play an important role in maintaining health.^1,5,7^ Interferons were the first cytokines that were used for therapeutic purposes as natural forms or recombinant forms in humans.^8^ In recent years, the therapeutic effects of various interferons have been identified on the treatment of various diseases, including hepatitis, cancers, autoimmune diseases, MS, and their importance in well-established therapeutic programs.^9-11^ The expression of type I and type III interferons usually increase significantly during viral infections, which indicates their role in controlling viral infections.^4^ In human, type I contains human interferon alpha (HuIFN-α; which has more than 12 encoding genes), HuIFN-β, HuIFN-ω, HuIFN-ε, and HuIFN-κ, which range in length from 172-165 amino acids. Their amino acid sequence homology is very close to each other^7,9,12^ (Figure 1). Type I interferons are known for their function in the immune system, but nevertheless play a role in immunization, control of cell proliferation, control of cancer cells, and the regulation of the acquired immune system.^13^ The distribution and expansion of interferon receptors in a wide range of cells indicate their wide and varied functions. All types of interferon-type I activate different cellular responses through binding to a common receptor containing two subunits IFNAR1 and IFNAR2.^14^ The affinity of the interferon type I to bind IFNAR1 and IFNAR2 receptors is different. Almost all species initially tend to form the IFNAR2-IFN binary structure, and then the IFNAR2-IFN-IFNAR1 triple structure will be created.^13^ It has been clearly demonstrated that the difference in the interferon affinity to the receptor, rather than the difference in the structure of interferons is causing different cellular messages and consequently different functions.^14^ The connection of HuIFN-β to IFNAR1 is 100-fold higher than the other Type I subunits.^14^ The binding of interferons of type I to its cell surface receptors activates the JAk1/STAT and TYK2 signaling pathway, which affects the expression of more than 2000 different genes with various biological functions.^10^ IFN-β is important in the treatment of MS and is one of the first and only developed drugs in the treatment of MS.^7,15^ Human Interferon beta (HuIFN-β) (UniProtKB-P01574), or interferon fibroblasts, is a glycoprotein with 166 amino acids of an approximate weight of 23 kDa, it is expressed only by a gene without intron and naturally has a glycosylation site on Asn80 in its structure. HuIFN-β is mainly produced by fibroblasts, but other cells, such as epithelial cells, dendritic cells and phagocytes, also play a role in its production and secretion.^9,16^ The secondary structure of HuIFAN-β and HuIFN-α2 are approximately the same, and in the IFNs I groups, only these two are naturally glycosylated.^1,17^ The secondary structure consists of five alpha helixes, consisting of A, B, C, D, E, and the distance between the regions is filled with a loop 2-28 residue (AB, BC, CD and DE ), (Figure 2).^17,18^ Helix A is parallel to B and anti-parallel to helixes C and E. The AB loop is larger than the other loops and is usually divided into three parts AB1, AB2 and AB3. In HuIFN-β, there is a disulfide bridge between the Cys31 in the loop AB and the Cys141 in the DE loop, both of which play an important role in connecting to the receptor. Also, Asn80 is naturally glycosylated, this sugar form a hydrogen bond with amino acids Asn86 (Helix C) and Gln23 (Helix A) in the other end. In addition to increasing solubility, it also results stability in the 3D structure of interferon.^17^ Today, various types of rHuIFN-β with different brands, such as Betaseron, Avonex and Rebif, are produced in prokaryotic and eukaryotic cells that are used to treat RRMS patients.^3^ The most significant difference between these three groups is in their specific activity, which is much higher in IFNβ-1a than in IFNβ-1b. The possible explanation for this, is the accumulation of IFN β-1b. IFN β-1b is produced in *E .coli*, unlike IFNβ-1a that is produced in eukaryotic cells (for example CHO). Therefore, it is not glycated, which leads to loss of its solubility, resulting in accumulation, loss of activity and a shorter half-life. As a result, IFN β-1b has a weak or ineffective connection to the receptor.^3^ Due to such a low activity, a very high dose level should be prescribed and this causes immunogenicity and production of antibodies against it, which is another disadvantage of the use of HuIFN β-1b. The importance of IFN-β in the treatment of MS has been revealed as well as its important therapeutic effects on rheumatoid arthritis, cancer and tumor have been studied.^9,15^ Therefore, considering the role and importance of HuIFN β in the treatment of autoimmune and inflammatory diseases such as MS and the treatment of viral infections, it is necessary to produce this drug in a better quality. In this regard, many studies have been done on improving the production, increasing the therapeutic effect, the quality and stability of recombinant IFN-β. Optimizing the culture medium, optimizing growth conditions and making changes in different areas of interferon mRNA, were causes to increase not only the expression but also the stability of HuIFN-β.^2,6^ The creation of the R27T mutation causes the formation of a new N-glycosylation site on the twenty-fifth amino acid (Asn25), which significantly increases the solubility and the half-life of rHuIFN-β.^6,19^ The V101F mutation also increases the expression of rHuIFNβ up to several times.^6,20^ Because R27 and V101 are in the region that are involved in the binding of HuIFN-β to their receptors, changes in these regions may be affected on interferon binding to their receptors. Despite the remarkable effect of these mutations to increase the solubility, half-life, activity and increasing the expression, so the effect of these mutations should be investigated for interferon binding to the receptor. In the present study, the effects of R27T and V101F mutations on the binding of wild type and mutated HuIFN-β 27-101 to IFNAR receptor were compared by using molecular docking.

**Table 1:** Comparison of Statistical Parameters of the Ramachandran diagram for wIFN and mIFNs

**Name**	**Mostfavouredregions [A,B,L]**	**Allowed regions [a,b,l,p]**	**Generously allowed regions [~a,~b,~l,~p]**	**Disallowed regions**	**Number of non-glycine and non-proline residues**	**Number of end-residues (excl.Glyand Pro)**	**Number of glycineresidues(shown as triangle)**	**Number of glycine residues**	**Total number of residues**
wIFNβ	145 92.4%	8 5.1%	3 1.9%	1 0.6%	157	2	6	1	166
mIFN 27	145 92.4%	8 5.1%	3 1.9%	1 0.6%	157	2	6	1	166
mIFN 101	145 92.4%	8 5.1%	3 1.9%	1 0.6%	157	2	6	1	166
mIFN27-101	145 92.4%	8 5.1%	3 1.9%	1 0.6%	157	2	6	1	166

A good quality model would be expected to have over 90% in the most favoured regions.

**Table 2:** The results of the negative energy connection (ΔGbind) for the four parameters of energy

	**Balanced (kcal/mol)**	**Electrostatic-favored (kcal/mol)**	**Hydrophobic-favored (kcal/mol)**	**Electro static + Van der Waals (kcal/mol)**
wIFN-IFNAR2	-1650	-1732.8	-1814.7	-316.4
mIFN27-IFNAR2	-1565	-1608.7	-1761.5	-292.6
mFN101-IFNAR2	-1652.8	-1734.2	-1815.3	-345.1
mIFN27-101-IFNAR2	-1563.9	-1624.8	-1916.5	-295.7
wIFN-IFNAR1	-1181.2	-1209.7	-1371.3	-229.7
mIFN27-IFNAR1	-1135	-1210.7	-1327.1	-224
mFN101-IFNAR1	-1126.6	-1125.4	-1211.5	-229.2
mIFN27-101-IFNAR1	-1124.8	-1151.7	-1262.8	-254.9

## Methods

Used from the crystalline structures of HuIFN-β (PDB ID: 1AU1), IFN-a 2a/IFNAR2 (PDB ID: 2HYM), HuIFNa2-IFNAR (PDB ID: 3se3, IFNAR2EC (PDB ID: 16vn) and IFNAR1 (PDB ID: 3s98) available on the RCSB server (https://www.rcsb.org/structure/1n6v). The simulation of arginine mutation into threonine in the twenty-seven amino acid (R27T), as well as the valine mutation in the phenylalanine mutation in the 101-amino acid (V101F) was developed on the Rosetta Backrub online server (https://kortemmeweb.ucsf.edu/backrub/cgi-bin/rosettaweb.py?query=index) based on the crystalline structure of HuIFN-β (PDB ID: 1AU1).^21^ Final evaluation of simulated structures on the server ( http://servicesn.mbi.ucla.edu/Verify3D/) verify3D was done ^22,23^ and Also, To review φ, ψ, and ω torsional angles in wild-type IFNβ structure and the changes of the angles possibly in mutant IFNβ, Ramachandran diagram or a [φ,ψ] plot were represented by SAVES web server (http://servicesn.mbi.ucla.edu/SAVES/). The comparison of the absolute surface area (ASA) of amino acids in the constructs created on the online server asaview (http://asaview.netasa.org).^24^ Also, the effect of mutations on protein structure and protein folds was investigated in HOPE (http://www.cmbi.ru.nl/HOPE) and SPDBV.^25,26^ Due to the structural similarity and receptor binding points between HuIFN-β and HuIFN-α2a, the combination of IFN-α 2a/IFNAR2 (PDB ID: 2HYM) as the primary template for combining HuIFN-β/IFNAR2 and the triple combination of HuIFNa2-IFNAR (PDB ID: 3se3) -β/IFNAR1 was used for molecular docking.

The molecular docking between HuIFN-β and the IFNAR receptor external region was performed using the online ClusPro2 protein-protein server (https://cluspro.org).^27^ For viewing, comparing and analyzing structures with PDB format, SPDBV v4.1, PyMOL v 0.99, and Chimera v 1.11, YASARA v 10.9.17 were used.

## Results

**Figure 3:**
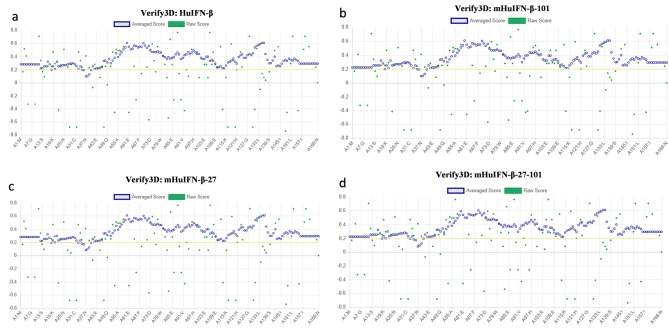
Analysis the simulated structures in verify3D. (a,b) The main structure of HuIFNβ and the simulated structure of mHuIFN-β-101 in Verify3D showed that 96.9% of the sequences had a score higher than or equal to 0.2 (average score ≥ 0.2). (c,d) The study of the simulated structure of mHuIFN-β-27 and mHuIFN-β-27-101 in Verify3D showed that 95.18% of the sequences had a score higher than or equal to 0.2 (average score ≥0.2).

**Figure 4:**
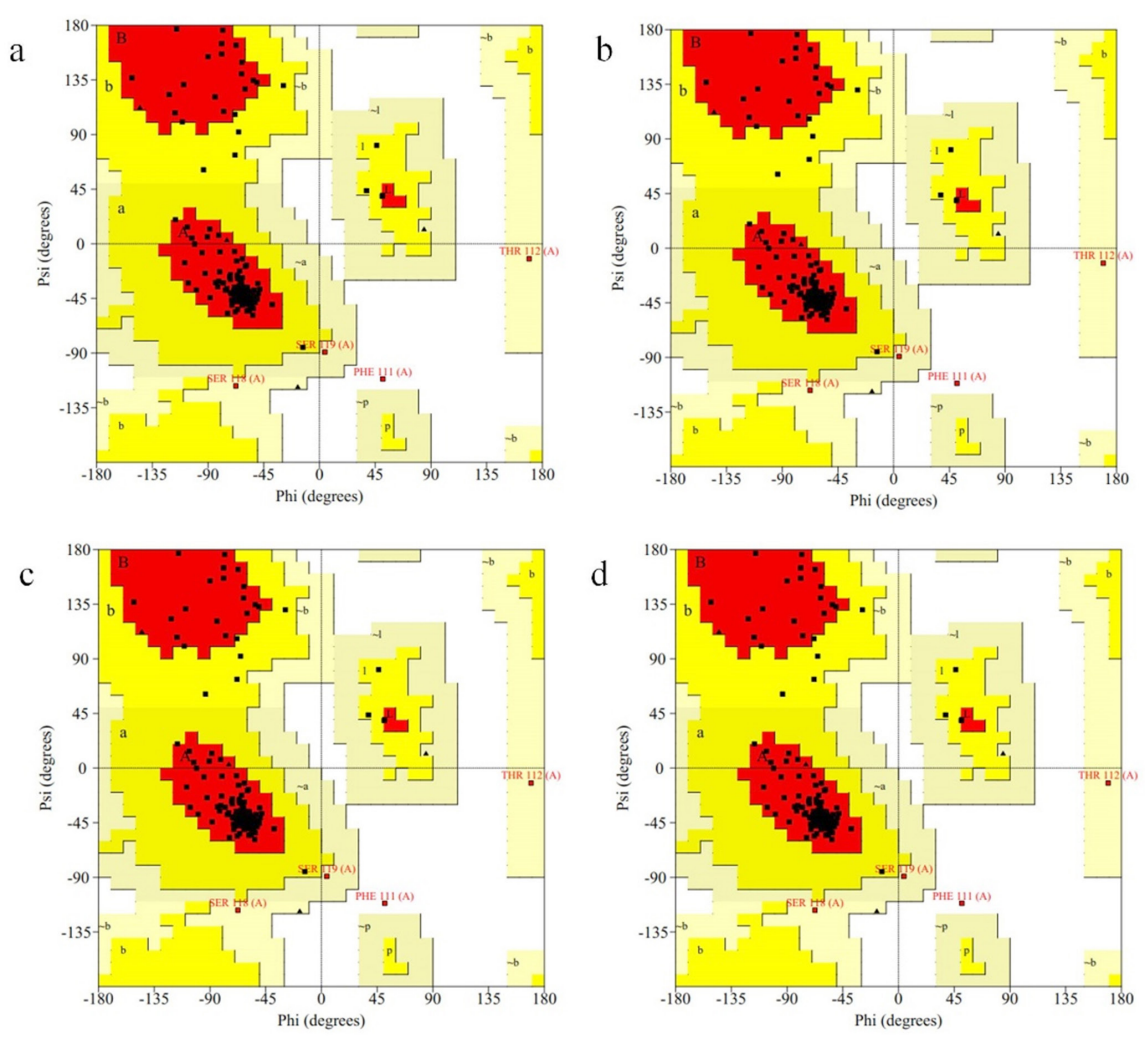
Ramachandran diagram for wild-type and mutant IFNβs are depicted. (**a)**Wild type (wIFN β), (**b,c)** and (**d)** mutant IFNβ respectively for mHuIFN-β-27, mHuIFN-β-101 and mHuIFN-β-27-101. The red regions were related to the residues in most favoured regions [A, B, L]. The yellow regions were related to the residues in additional allowed regions [a,b,l,p]. The pea green regions were related to the residues in generously allowed regions [~a,~b,~l,~p]. The white regions were related to the residues in disallowed regions.

**Figure 5:**
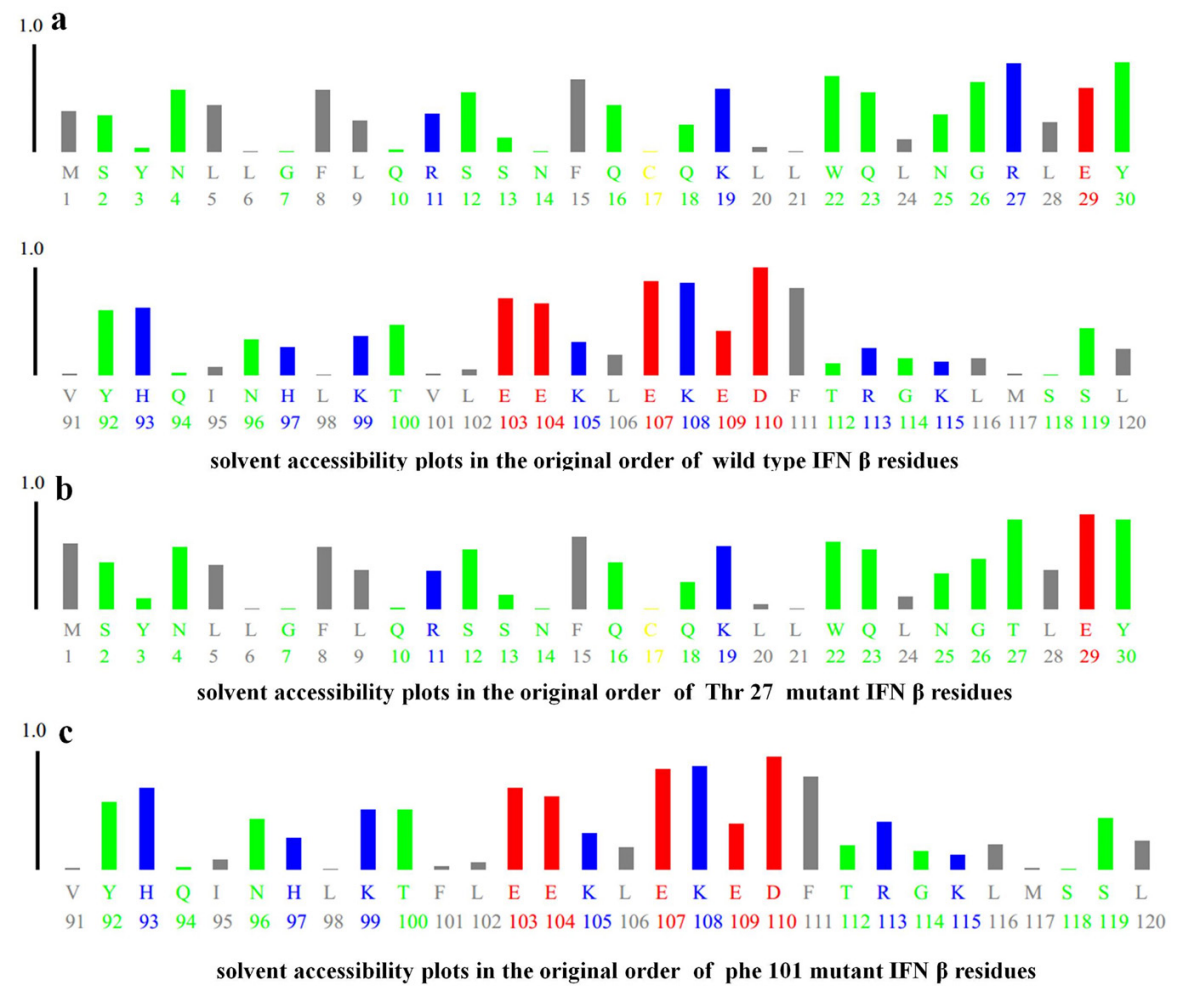
Effect of mutation on access to solvent of amino acid (ASA). (a) Position and access to the R27 and V101 solvent and surrounding amino acids in wIFN. (b) in mIFN, where the new amino acid (T27) changes the access to solvent of some amino acids, and this occurs in E29, G26 and M1 More evident. (c) The V101F substitution in mIFN does not have much effect on the surrounding amino acids. V101 and Phe101 are both in the inner part of the molecule and their access to the solvent is very low.

**Figure 6:**
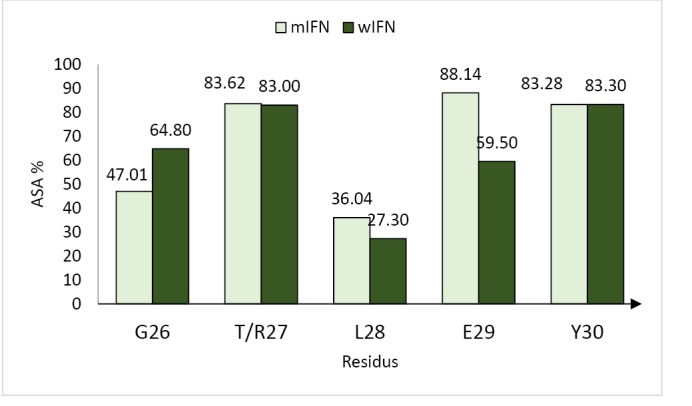
The effects of R27T substitution on access to solvent of adjacent amino acid.

**Figure 7:**
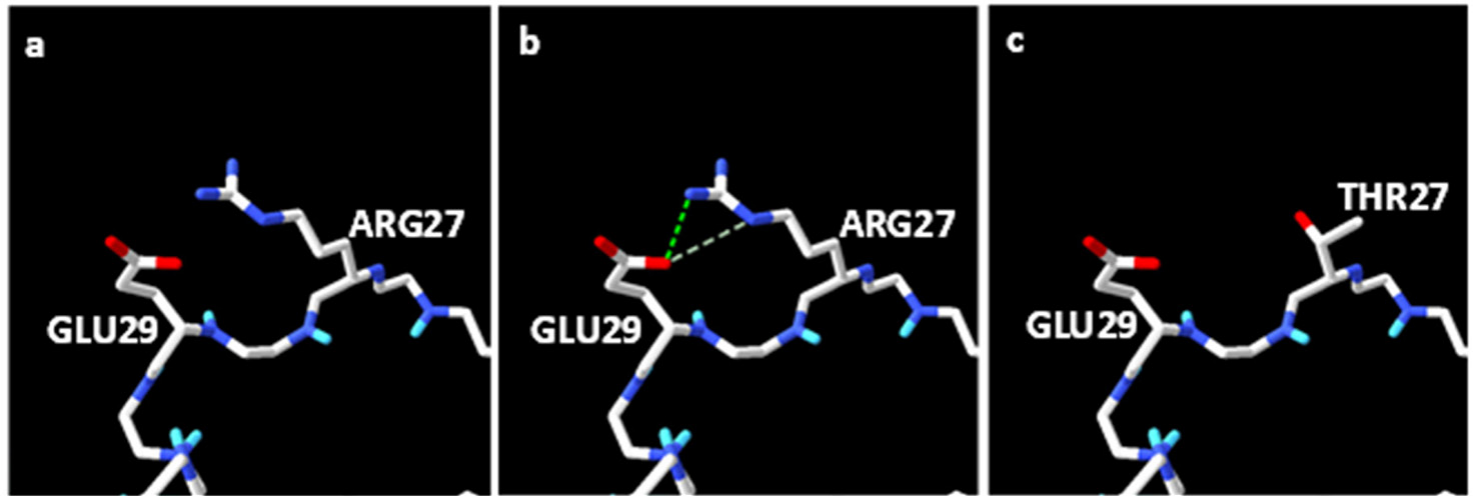
Changes caused by the replacement of R27T. (a) The position of the amino acid ARG27 adjacent to the GLU29 amino acid. (b) The two amino acids ARG and GLU are able to form a hydrodynamic relationship with respect to distance and position. (c) By replacing the R27T, the side chains are different and the position and the two amino acids THR and GLU differ, so that the hydrodynamic relationship between them is not created (provided with the help of the SPDBV software).

**Figure 8:**
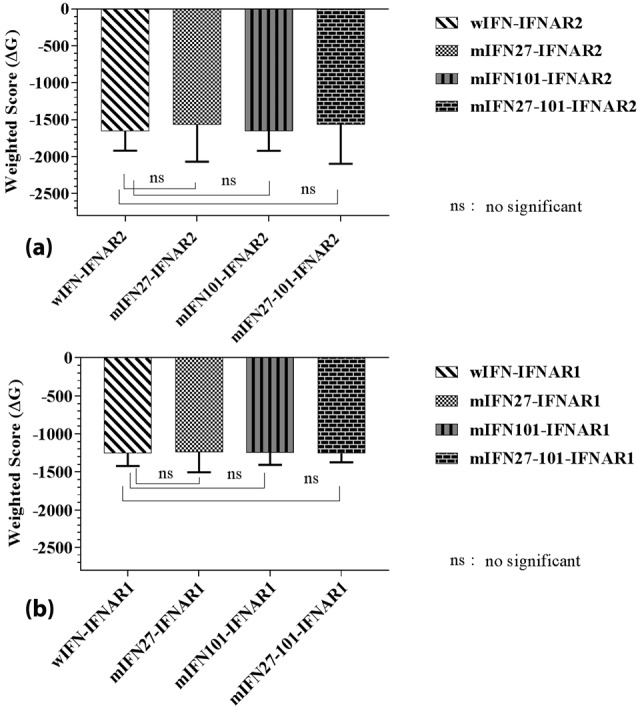
The comparison of ΔG from docking molecules. A)- The results of the comparison of the GΔ between wIFN/IFNAR2 and the mIFN/IFNAR2 complex, which indicates that the variation of GΔ is not significant. B)- The results of the comparison of the GΔ complex between wIFN/IFNAR1 and the mIFN/IFNAR1 complex, indicating that the variation of GΔ is not significant.

The mHuIFN-β-27, mHuIFN-β-101 and mHuIFN-β-27-101 structures were simulated on the basis of the crystalline structure HuIFN-β (PDB ID: 1AU1) in the Rosetta Bakrub server, and the structures with the highest score (the most negative ΔG) were selected. The study of simulated structures in verify3D showed that more than 95% of the residue in all simulated structures had scores higher than or equal to 0.2 (average score> 0.2) (Figure 3). The Ramachandran diagram showed the created mutation has not changed at the angles φ, ψ and ω , in the wild-type IFNβ Arg27 (φ = - 29.14, ψ = 130.33, and ω = 179.14), and Val101 (φ = - 65.69, ψ = - 37.75, and ω = 179.18) and in the mutant IFNβ Thr27 (φ = - 29.14, ψ = 130.33,and ω = 179.14) and Phe101 (φ = - 65.69, ψ = - 37.75, and ω = 179.18), and these angles are the same in both the natural and mutated structures (Figure 4; Table 1). Comparison of ASA or accessible to solvent of amino acids of wild and mutated structures showed that, replacement of an amino acid with different side chains in size and type, causing increased and decreased the ASA of amino acids (Figures 5 and 6). HOPE’s online software showed that the replaced amino acid with different side chains in type, size and specification may causes changes in hydrogen bonds and interactions with other molecules (receptors), which is also visible in SPDBV (Figure 7). The results of protein-protein molecular docking were compared with the IFN-a 2a/ IFNAR2 (PDB ID: 2HYM2) and the triple combination of HuIFNa2-IFNAR (PDB ID: 3se3) generally analyzed in four energy parameters (Table 2). The comparison of the values of the negative binding energy (ΔGbind) obtained from protein-protein molecular docking between IFNAR receptor and HuIFN-β, mHuIFN-β-27, mHuIFN-β-101 and mHuIFN-β-27-101 ligands was not significantly different and there was no significant difference between them (*P* value > 0.9999) (Figure 8), with regard to these results, it can be concluded that the mutations produced do not have a negative effect on the composition of mHuIFN-β/IFNAR.

## Discussion

The creation of these mutations (R27T) and (V101F) have already been studied in order to improve the quality, stability and enhancement of HuIFN-β expression. On the other hand, the therapeutic effects of these mutations are also remarkable in such a way to not only improve the half-life of the mHuIFN-β in the body, but also decrease its immunogenicity.^6,19,20^ The mutation (R27T) produces a new glycosylation signal on rHuIFN-β and glycosylation of the Asn25 amino acid. The glycosylation of the Asn25 in the binding of rHuIFNβ-1a to the receptor not only does not inhibit, but also by creating a new hydrogen bond relationship between Asn25-linked glycan and Thr44, Asn51 of receptor IFNAR2, may lead to creating a more stable compound between the mHuIFN-β-27/IFNAR2.^19^ Goelz et al and also Key et al showed that the V101F mutation increases the expression and the antiviral property of mHuIFN-β several times in comparison to the wild type interferon.^6,20^ The important amino acids in the binding of HuIFN-β to IFNAR2 receptor are in regions A2, AB1, AB2, E and to IFNAR1 receptors in regions B, C2, DE.^28^ R27 is located in the AB1 region and V101 is located in the C2 region that is important for binding of HuIFN-β to its IFNAR receptor. Therefore, these changes in general may impact on the binding of HuIFN-β to its own receptor, which may ultimately affect its function and therapeutic outcomes. In this study, the effects of these mutations on access to amino acid solvent and its effects on protein structure and ultimately the effect of these mutations on the binding of rHuIFNβ-1a to the common receptor of IFNs type I (IFNAR) were studied.

The results of the Verify3D server showed that the simulated structures have high scores and more than 95% of the residues in all structures had scores higher than or equal to 0.2 (average score ≥ 0.2), which is very close to the original structure (PDB ID: 1AU1) HuIFN-β, which has 96.99% of the sequences with a score higher than or equal to 0.2 (average score ≥ 0.2) and were used to continue the study (Figure 3). The Ramachandran diagram or a [φ,ψ] plot showed the folding of mutant IFN beta is correct. The calculated torsional angles revealed that the substitutions of R27T and V101F could not have an effect on the correct folding of IFNβ and these mutations did not disturb angles in comparison with the wild-type IFNβ. ASA or accessible to solvent of amino acids in a protein has so many important applications. Identification of the level of accessions of amino acids helps to identify potential active sites in a protein. Online software asaview is an algorithm, application, and database with access to solvent of amino acids in protein structure that easily calculates the accessibility of an amino acid solvent. This software determines the amino acids that are available on the surface, as well as the amino acids that are present in the inner part. It then provides a graph of the topological distribution of amino acids. Comparison of access to solvent of amino acids from wild and mutated structures showed that due to the replacement of amino acids with different lateral chains with different in sizes and types, they cause a huge change in ASA (increase and decrease) of amino acid (Figure 6). Both the T and R amino acids have solvent accessibility a higher than 80%, and their side chains are well accessible due to their position on the outer surface of the molecule (Figure 5), but the amino acids F and V are both inside and hidden. As their solvent access is less than 3%, their access to the molecular level is much lower, and the replacement (V101F) has not significantly changed the availability of solvent to the surrounding amino acids (Figure 5). The replacement of T at position 27 caused a change in the solvent accessibility of adjacent amino acid, especially at E29, whose solvent availability increased from 59.5% to 88%, increasing the availability of solvent in amino acid L28 from 27.3% to 36.04% and the reduction of solvent access in amino acid G26 from 64.8% to 47.01% (Figure 6). These amino acids contribute to the IFN binding to the receptor, which the mutation may be affected in the IFN binding to its receptor. HOPE is a fully automated program that analyzes the structural and functional effects of point mutations. HOPE collects information from a wide range of information sources, such as calculations in 3D protein coordinates using the WHAT IF server service, data sequences from UniProt and DAS service (Dense Alignment Surface).^26^ At HOPE, the homology of the models is done with (YASARA’s; http://www.yasara.org/). The data obtained are stored in a database. The final results of analyzing of the effects of the mutation in the structure and protein function are then used. HOPE creates a report with text, figures and animations that is easy for researchers to understand.^26^

The results obtained from HOPE showed that in the substitution of R27T, each amino acid has its own specific size, charge, and hydrophobicity-value. But these properties are varied in the substituted residues. The original residue is bigger than the mutated residue. The wild-type residue is less hydrophobic than the mutated residue. Naturally, there is a hydrogen bond and a salt bridge between the amino acids R27 and E29. The difference in charge and size between the amino acids of the T and R proteins interferes with the interaction. HOPE also showed that the residue at this position is not protected, and other residue, as well as T has been observed at this site in other homologous sequences. So the mutation has probably been not damaged the protein. The results obtained from HOPE in relation to the mutation V101F showed the substituted residues are different in properties. The mutant residue is bigger than the wild-type residue. The wild-type residue was buried in the core of the protein and F residue probably will not fit. Another residue type was observed more often in this position in other homologous sequences and the wild-type residue is not conserved at this position. But since the homologous region is not observed, this mutation is likely to be harmful. The HOPE revealed that the V101 is located in the region, which is important to contact of interferon with other molecules. This replacement may cause damage to these contacts and also affect the function of the protein too.

For molecular docking, the crystalline structure of the combination of HuIFN-β/IFNAR2 and HuIFN-β/IFNAR1 is not present. There is a very high similarity between the HuIFN-β structure and the HuIFN-α2a structure and their binding points to the receptor, so the combination of IFN-a 2a/IFNAR2 (PDB ID: 2HYM) and the triple combination of HuIFNa2-IFNAR (PDB ID: 3se3) were used as a preliminary template to determine the approximate position and location of rHuIFN-β on the receptors.^29,30^

Due to the specificity of the important residues in the binding of HuIFN-β to the receptor and the domains involved in a binding^17,30^ between the structures of mHuIFN-β-27, mHuIFN-β-101, mHuIFN-β-27-101 and HuIFN -β with the IFNAR receptor by using the online Cluspro 2.2 software molecular docking done. The ClusPro (https://cluspro.org) is a server that is widely used for protein-proteins docking. Working with the server is simple and easy, requiring only two files containing two PDB format receptor and Ligand sequences. It also has a number of advanced options that can be used to accelerate and modify the docking process. This server performs the computational steps as follows:

(1) rigid body docking by sampling of billion combinations,(2) root-mean-square deviation (RMSD) clustering of 1000 structures with the lowest energy produced to find the largest clusters that represent the most likely models of the combination, (3) filtration selected structures with energy minimization. At the end of the results, the multi parameters of energy are provided in 10 models for each docking, and the group with highest demographic and a minimum value of energy and the highest score that can be most likely combined.^27^ Based on the molecular Docking results, those models whose geometric structures were similar were grouped according to RMSD criteria: Each group included structures that differed by a maximum magnitude of RMSD of 9A˚. Similar models of the molecular docking results were selected with the highest number in one set and the minimum of energy in approximately the same location in comparison with IFN-a 2a/IFNAR2 (PDB ID: 2HYM), IFN-β-IFNAR1 (PDBID): 3wcy) and a combination of HuIFNa2-IFNAR (PDB ID: 3se3). They were evaluated in four sets of different energy parameters including Balanced, Electrostatic-Favored, Hydrophobic-Favored and Electro Static + Van der Waals. The comparison of the binding energy (ΔGbind) obtained from molecular docking between the structures of mHuIFN-β-27, mHuIFN-β-101, mHuIFN-β-27-101 and wHuIFN-β with IFNAR receptors does not have any significant difference between the mutated and the wild type interferons binding into the IFNRA receptor (*P* > 0.9999).

## Conclusion

It can be said that the creation of the mutations R27T and V101F in HuIFN-β, despite the presence of these points in important areas and result of ASA and HOPE, does not interfere with the receptor binding. In previous studies, it has been shown that these mutations increase not only the quality and efficacy of the rHuIFN-β but also its expression. Therefore, it seems that the creation of this mutation is very suitable for the rHuIFN-β structure and improves the quality and quantity of rHuIFN-β without any interfering with its binding to IFNAR receptor.

## Ethical Issues

This work is a bioinformatic and computational study, therefore there was no need to any ethical confirmation in order to be performed.

## Conflict of Interest

The authors declare that they have no conflict of interest.

## Acknowledgments

This article is based on the Ph.D. thesis and a research project number of 92042856. Hereby, Department of Biology at University of Isfahan are sincerely appreciated for providing facilities and equipment for this study. Thanks to Dr. Ganjali Khani and Mr. Dehbashi, who that helped me to do this research.
